# Diagnostic performance of ^18^F-PSMA-1007 PET/CT in patients with biochemical recurrent prostate cancer

**DOI:** 10.1007/s00259-018-4089-x

**Published:** 2018-07-20

**Authors:** Kambiz Rahbar, Ali Afshar-Oromieh, Robert Seifert, Stefan Wagner, Michael Schäfers, Martin Bögemann, Matthias Weckesser

**Affiliations:** 1 Department of Nuclear Medicine, University Hospital Münster, Albert-Schweitzer-Campus 1, 48149 Münster, Germany; 2 Department of Nuclear Medicine, Inselspital, University Hospital Bern, Bern, Switzerland; 3 Department of Nuclear Medicine, Heidelberg University Hospital, Heidelberg, Germany; 4 Department of Urology, University Hospital Münster, Münster, Germany

**Keywords:** Prostate cancer, PSMA-1007, Biochemical relapse

## Abstract

**Purpose:**

The introduction of ligands targeting prostate-specific membrane antigen (PSMA), especially ^68^Ga-PSMA-11, has changed the management of patients with prostate cancer (PCa). ^18^F-Labelled ligands can be produced in larger amounts and therefore can improve availability for a larger group of patients. The aim of this study was to evaluate the diagnostic performance of the recently introduced ^18^F-PSMA-1007 in patients with recurrent PCa.

**Methods:**

This retrospective analysis included 100 consecutive patients with biochemical relapse (mean age 68.75 ± 7.6 years) referred for PSMA PET/CT. Whole-body PET/CT imaging (from the lower limbs to the skull) was performed in all patients 120 min after injection of 338 ± 44.31 MBq ^18^F-PSMA-1007. Prostatectomy, radiation beam therapy of the prostate bed and androgen-deprivation therapy had been performed in 92%, 45% and 27% of the patients, respectively. Radiation beam therapy of the prostate bed had been performed in addition to surgery in 38 patients (38%) and 10 patients (10%) had received all three therapy modalities. The probability of a ^18^F-PSMA-1007 PET/CT scan suggestive of pathology was compared with the Gleason score (GS) and PSA level.

**Results:**

Of the 100 patients, 95 (95%) showed at least one pathological finding on ^18^F-PSMA-1007 PET/CT. The overall median PSA level was 1.34 ng/ml (range 0,04–41.3 ng/ml). The rates of pathological scans were 86%, 89%, 100% and 100% among patients with PSA levels ≤0.5, 0.51–1.0, 1.1–2.0 and > 2.0 ng/ml, respectively. The median GS was 7 (range 5–10). The majority of patients (70) with a GS available had a score in the range 7–9. The rate of pathological scans in these patients was 93% (65/70). The median SUV_max_ values of the pathological findings were 10.25, 14.32, 13.16 and 28.87 in patients with PSA levels ≤0.5, 0.51–1.0, 1.1–2.0 and >2.0 ng/ml, respectively. The median SUV_max_ in patients with a PSA level of >2.0 ng/ml was significantly higher than in all other PSA groups.

**Conclusion:**

^18^F-PSMA-1007 PET/CT can detect recurrent PCa in a high percentage of patients with biochemical relapse. The probability of a pathological ^18^F-PSMA-1007 PET/CT scan seems to be high even in patients with a low PSA level ≤0.5 ng/ml, and this may have a significant impact on the management of this relevant group of patients.

## Introduction

Prostate cancer (PCa) is the most frequent tumour disease in men worldwide [1]. Biochemical relapse is frequent after initial curative treatment, especially in patients with high-risk PCa. Conventional imaging with, for example, computed tomography (CT) and magnetic resonance imaging (MRI), are insufficient in detecting tumour lesions due to their low sensitivity and specificity. During recent years targeted imaging of prostate-specific membrane antigen (PSMA), also known as glutamate carboxypeptidase II, *N*-acetyl-α-linked acidic dipeptidase I or folate hydrolase, has opened a new chapter in the diagnostic management of patients with PCa [2–6]. PSMA is a type II transmembrane glycoprotein that is strongly overexpressed in PCa cells. The level of PSMA expression rises with increasing tumour dedifferentiation and is higher in hormone-refractory cancers [7–9].

Because of the larger activity amount of cyclotron-produced ^18^F compared with the limited activity of ^68^Ga derived from ^68^Ge/^68^Ga generator elution, and its longer half-life and higher physical spatial resolution, ^18^F has advantages over ^68^Ga [10]. Furthermore, the very low urinary activity in ^18^F-PSMA-1007 PET/CT scans seems to be another advantage, which makes this new imaging pharmaceutical highly interesting for the differentiation of lymph node metastases of recurrent PCa from urinary activity in the ureter or for the differentiation of local relapse from the urinary bladder [11].

The first clinical experience in single cases or case series with ^18^F-PSMA-1007 has shown the potential of this new radiopharmaceutical in patients with PCa [11–14]. The aim of the present study was to evaluate the performance of ^18^F-PSMA-1007 in detecting PCa recurrence or metastases in patients with biochemical relapse.

## Materials and methods

### Patients

From October 2017 to May 2018, 212 patients were examined with ^18^F-PSMA-1007 PET/CT as part of clinical routine. Of these patients, 100 were referred for the detection of recurrent PCa. The patients received primary therapy for PCa a median of 44.3 months before PSMA imaging (range 2–314 months). In seven patients, the exact time of the first therapy for PCa was not available. Some of the patients had undergone CT, MRI or bone scan before PSMA imaging. However, a systematic comparison of these data was not possible since the intervals were highly variable and different procedures had been performed. All other imaging studies performed before PET/CT had been negative. Of the 100 patients, 28 had been analysed previously in a dual time scanning analysis [14].

**Fig. 1:**
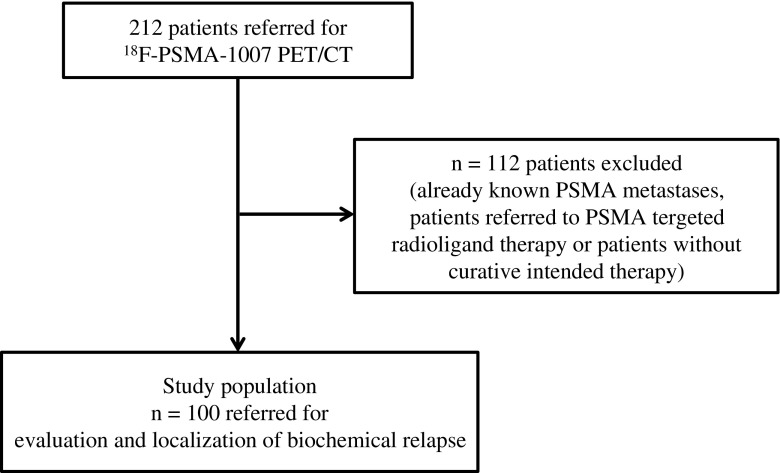
Flow chart of patient selection

Patient characteristics are given in Table 1. Patients with no primary therapy with curative intent or patients referred for PSMA radioligand therapy were not included in the current analysis (Fig. 1). Prostatectomy, radiation beam therapy and androgen-deprivation therapy had been performed in 92%, 45% and 27% of the patients. Radiation beam therapy in addition to surgery had been performed in 38 patients (38%) and 10 patients (10%) had received all three therapy modalities. All patients received detailed information about the imaging procedures and provided signed informed consent according institutional guidelines.

**Table 1:** Characteristics of the 100 included patients

Characteristic	Value
Age (years)	
Mean ± SD	68.75 ± 7.6
Median (range)	70.44 (47.36–85.82)
Administered activity (MBq)	
Mean ± SD	338.02 ± 33.31
Median (range)	334 (200–412)
Gleason score^a^	
Mean ± SD	7.5 ± 1.01
Median (range)	7 (5–10)
PSA level at PET (ng/ml)	
Mean ± SD	3.36 ± 6.11
Median (range)	1.34 (0.04–41.3)
Prior therapies, *n* (%)	
Prostatectomy	92 (92)
Radiation beam therapy to prostate bed	45 (45)
Androgen-deprivation therapy	27 (27)

*SD* standard deviation, *PSA* prostate-specific antigen

^a^In 76 patients; data from 24 patients missing

### Imaging procedures and preparation of ^18^F-PSMA-1007

^18^F-PSMA-1007 was produced in a GE TracerLab MX synthesizer according to the one-step procedure described by Cardinale et al. and standard operation procedures described previously [14, 15] including sterile filtration of the final batch solution. ^18^F-PSMA-1007 precursor, cassettes and reagents for the synthesis of ^18^F-PSMA-1007 as well as the synthesis sequence for fully automatic production with a GE TracerLab MX module were obtained from ABX GmbH (Radeberg, Germany). The final injection solution of the ^18^F-PSMA-1007 batch was clear, colourless and particle-free, and had a mean radiochemical purity of 96.5 ± 1.1% (range 95–99%) as determined by high-performance liquid chromatography. Unreacted ^18^F-fluoride or ^18^F-fluoride resulting from compound cleavage was not detected by thin-layer chromatography. The pH of the batch solution ranged from 5.9 to 7.8 and the endotoxin content was <5.0 endotoxin units/ml. The concentrations of ethanol and dimethyl sulfoxide (DMSO) as residual solvents were measured by gas chromatography (ethanol 29.6–31.6 mg/ml; DMSO 0.25–0.68 mg/ml). The osmolality ranged from 1,110 to 1,300 mOsmol/kg.

Patients received 4 MBq/kg body weight with a maximum of 400 MBq per patient (mean injected activity 338 ± 44.31 MBq). Scanning was performed 120 min after injection from the lower limbs to the skull. Patients were asked to empty their bladder before the scan. Images were acquired with a scan time of 3 min per bed position on a Siemens mCT scanner (Siemens Healthcare, Knoxville, TN). Images were reconstructed using the standard software provided by the manufacturer. For attenuation correction, a low-dose CT scan was performed in accordance with the PET imaging. Contrast-enhanced CT of the abdomen and pelvis was only performed if no recent CT or MRI scans were available.

### Image analysis

Coregistered images were analysed using *syngo*via software, version: VB20A (Siemens Healthcare). According to institutional procedures all scans were analysed by two board-certified nuclear medicine physicians and radiologists at an interdisciplinary conference for reporting. Absence of morphological changes was regularly found and were classified as bone metastases and local recurrence. The sensitivity of CT for these types of metastases is known to be limited, and thus high focal tracer uptake in these areas was considered suggestive of bone metastases or local recurrence despite a lack of morphological correlation. In lesions characteristic of PCa, volumes of interest were placed on the plane with highest uptake, and maximum standardized uptake values (SUV_max_) were measured and documented. Focal tracer uptake above local background in morphologically visible lesions on CT was considered as PSMA-positive. Any visible PCa lesions were analysed unless the patients had more than three lesions, in which case, a maximum of three lesions were analysed. This kind of selection avoids overestimation of SUVs as otherwise dominant lesions would be preferentially selected. Typical pitfalls such as PSMA uptake in sacral and coeliac ganglia or in the stellate ganglia were frequently observed but were not considered pathological [16].

### Statistical analysis

SPSS Statistics 25 (IBM Inc. Armonk, NY, USA) was used for statistical analysis. Descriptive statistics are absolute and relative frequencies, mean or median and standard deviation or range, were used to characterize the study population. For subgroup analysis, patients were divided into four groups depending on their prostate-specific antigen (PSA) level: ≤0.5, 0.51–1.0, 1.1–2.0 and >2.0 ng/ml. The Mann-Whitney test was used to evaluate the significant differences of the median SUV_max_ (of the lesion with maximum uptake from each patient) between PSA groups. A *P* value <0.05 was considered significant and noticeable.

## Results

**Fig. 2:**
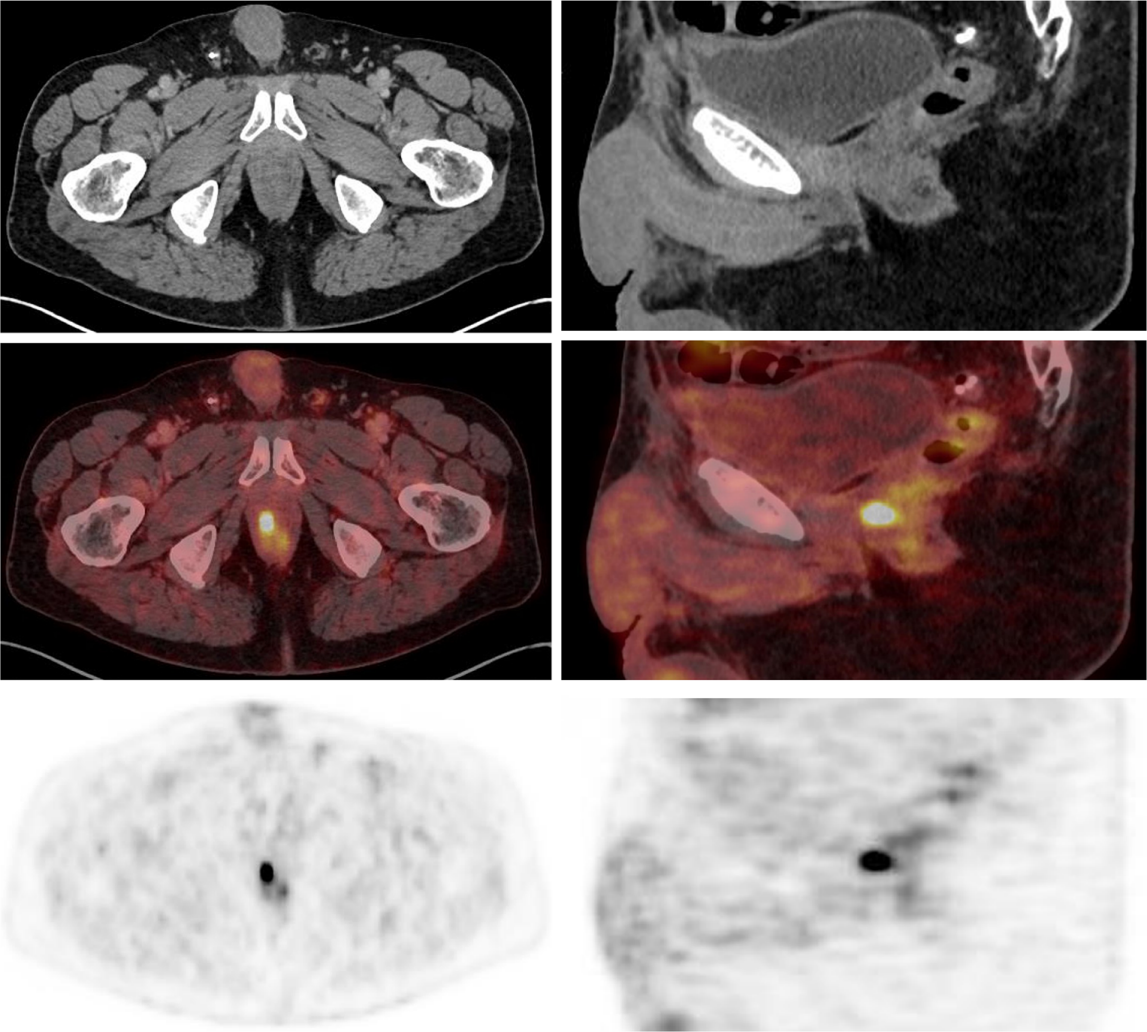
^18^F-PSMA-1007 PET/CT imaging (*left* axial images, *right* sagittal images) in a 72-year-old patient with biochemical relapse (PSA level 0.86 ng/ml) and with a Gleason score of 7b (4 + 3). The images show focal right-sided tracer uptake in the prostate bed

**Fig. 3:**
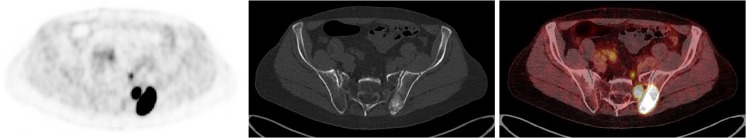
^18^F-PSMA-1007 PET/CT imaging (axial images: *left* PET, *centre* CT, *right* fused PET/CT) in a 73-year-old patient with biochemical relapse (PSA level 1.05 ng/ml) after radical prostatectomy and prior antiandrogen therapy and radiation to prostate bed (Gleason score not available). The images show bone metastases in the left pelvis and in the adjacent os sacrum. An additional presacral lesion is seen on the left side suggestive of lymph node metastasis

**Fig. 4:**
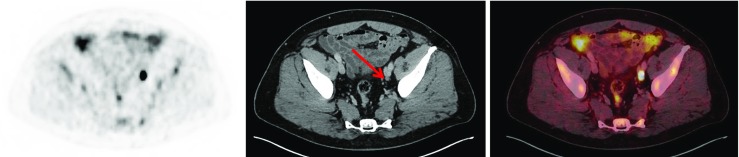
^18^F-PSMA-1007 PET/CT imaging (axial images: *left* PET, *centre* CT, *right* fused PET/CT) in a 59-year-old patient with biochemical relapse (PSA level 0.54 ng/ml) after radical prostatectomy and with a Gleason score of 7b (4 + 3). Images show a 5-mm lymph node lesion (*arrow*) with high tracer uptake along the left iliac artery

The analysis included 100 consecutive patients (mean age 68.75 ± 7.6 years) who were referred for ^18^F-PSMA-1007 PET/CT for evaluation and localization of biochemical relapse. None of the patients showed any kind of adverse event or clinically detectable pharmacological effect following injection of ^18^F-PSMA-1007. In 95 of the 100 patients (95%) at least one lesion suggestive of PCa was detected on ^18^F-PSMA-1007 PET/CT. The patient-based sensitivity was therefore 95% (95% confidence interval 91–99%). In total, 213 lesions characteristic of PCa (37 local relapses, 107 lymph node metastases, 67 bone metastases and 2 soft tissue metastases) were detected in 95 patients, and in 29 patients relapse was exclusively lymph node metastases, in 16 patients exclusively bone metastases and in 15 patients exclusively local relapse. Table 2 presents the average SUV_max_ of all included lesions. Examples of a bone lesion, a lymph node and a local relapse are given in Figs. 2, 3 and 4.

**Table 2:** Average SUV_max_ of PCa lesions of all types

Lesion type	No. of lesions	SUV_max_			
		Mean ± SD	Minimum	Maximum	Median
All lesions	213	21.52 ± 29.8	2.49	248.28	11.45
Local relapse	37	18.29 ± 23.78	3.87	137.94	10.64
Lymph node metastases	107	26.03 ± 34.51	2.59	248.28	16.1
Bone metastases	67	16.57 ± 23.59	2.49	136.18	7.5
Soft tissue metastases	2	6.82 ± 3.7	4.19	9.46	6.8

*SD* standard deviation, *PCa* prostate cancer

**Fig. 5:**
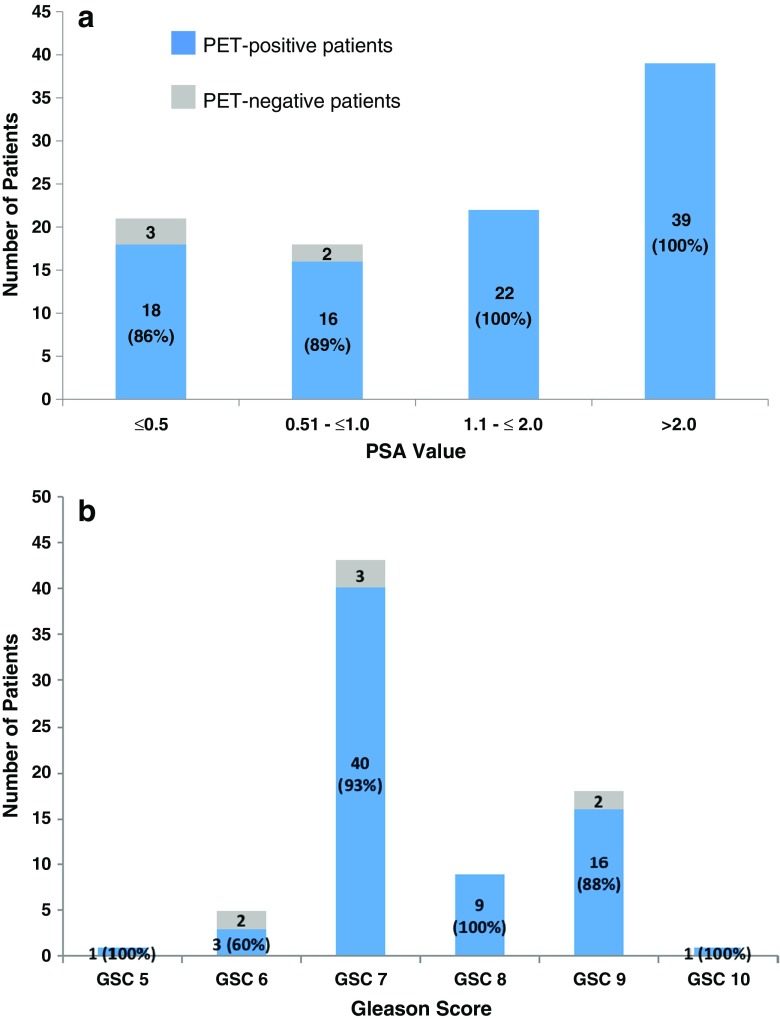
Probability of pathological ^18^F-PSMA-1007 PET/CT in relation to PSA level (**a**) in 100 patients and in relation to Gleason score (**b**) in 76 patients. *Blue columns* show the number and percentage of patients with a pathological ^18^F-PSMA-1007 PET/CT scan. *Grey columns* show the number and percentage of patients with a negative PET scan

Of patients with a PSA level ≤2.0 ng/ml, 92% were PET-positive. The rates of pathological ^18^F-PSMA-1007 PET/CT scans in relation to PSA level and Gleason score (GS) are presented in Fig. 5. In total, ^18^F-PSMA-1007 PET/CT was negative in five patients, three with a PSA level ≤0.5 ng/ml and two with a PSA level in the range 0.51–1.0 ng/ml.

The majority of patients (70) with a GS available had a score in the range 7–9. The detection rate in these patients was 93% (65/70). The median SUV_max_ values of the pathological findings in patients with PSA levels ≤0.5, 0.51–1.0, 1.1–2.0 and > 2.0 ng/ml were 10.25 (range 3.98–48.99), 14.32 (range 4.36–38.61), 13.16 (range 3.14–136.18) and 28.87 (range 5.03–248.27), respectively. The median SUV_max_ in patients with a PSA level >2.0 ng/ml was significantly higher than that in patients in the other PSA groups (>2.0 ng/ml vs. ≤0.5 ng/ml, *P* = 0.001; vs. 0.51–1.0 ng/ml, *P* = 0.005; vs. 1.1–2.0 ng/ml, *P* = 0.027). There were no significant differences among the other PSA groups.

## Discussion

PSMA-targeted imaging has achieved a leading role in the management of PCa patients during recent years overcoming the challenging lack of sensitivity and specificity of conventional imaging modalities. In the present study the performance of ^18^F-PSMA-1007 in detecting PCa lesions in patients with biochemical relapse was evaluated.^18^F-PSMA-1007 PET/CT scans in 100 consecutive patients referred for evaluation and localization of biochemical relapse were retrospectively analysed. Of all the patients included in this analysis, 95% showed at least one lesion with characteristics suggestive of PCa on ^18^F-PSMA-1007 PET/CT. A recent case series of 12 patients revealed a detection rate of 75% [17], which is lower than in the present study, but the detection rate found in that study might have been due to the small number of patients. In the study mentioned, 62% of the patients who had a PSA level ≤2.0 ng/ml had a pathological scan, whereas in our study 92% of the patients (56/61) with a PSA level of ≤2.0 ng/ml had a pathologic scan.

The PET-positive rate in the present study was higher than the detection rate in a study investigating the performance of ^68^Ga-PSMA-11 PET/CT in 1007 patients with biochemical relapse (the largest group investigated so far) [3]. In that study the detection rate was 79.5% in patients with a median PSA level of 2.2 ng/ml compared with 95% in the present study and a median PSA level of 1.34 ng/ml. In another study also using ^68^Ga-PSMA-11, the detection rate was 89.5% in 248 patients with biochemical relapse and a median PSA level of 1.99 ng/ml [18]. In that study, the detection rate in patients with a PSA level <0.5 ng/ml was 57.9% (11/19) compared with a rate of 86% (18/21) in our study. Although the patient groups are not directly comparable (e.g. different proportions of patients with previous surgical treatment), our results could indicate that ^18^F-PSMA-1007 has a higher sensitivity than ^68^Ga-PSMA-11. However, further studies including more patients are mandatory to further analyse the sensitivity of ^18^F-PSMA-1007.

In the present study, local relapse was detected in 37% of patients (37/100), which is considerably higher than found in a recent study using ^68^Ga-PSMA-11 in which local relapse was detected in 4% of patients (13/319) [2]. Our results suggest that ^18^F-PSMA-1007 might be superior to ^68^Ga-PSMA-11, especially in patients with a low PSA level. However, this suggestion must be further evaluated in a head to head comparison, which is not yet available. Furthermore, it is well known from ^68^Ga-PSMA-11 and ^18^F-PSMA-1007 studies that PCa lesions are shown with better contrast and higher tracer uptake after longer uptake times (e.g. 3 h rather than 1 h after injection) [2, 6, 14]. This indicates that imaging with ^18^F-PSMA-1007 is probably more advantageous at 2 h than at 1 h after injection, which has become common with ^68^Ga-PSMA-11 at most centres. The longer half-life and higher injected activities allow high-quality delayed images, higher lesion uptake and superior clearance of background activity. The higher detection rate may therefore be because of the superior differentiation of ureter and bladder activity from local recurrence and locoregional lymph node metastasis [11, 14]. The confidence in diagnosing local recurrence is thus enhanced. Particularly in patients with a low PSA level, radiotherapy of local recurrences may induce secondary complete remission. A reliable imaging procedure for use in patients with increasing but still low PSA levels is thus of the utmost clinical relevance. The nonsignificant differences in median SUV_max_ among the subgroups of patients with a PSA level ≤2.0 ng/ml underlines the high detection rates revealed in the present analysis in patients with a very low PSA level (e.g. ≤0.5 ng/ml). A lower PSA threshold level can thus not be recommended since tumour lesions can be visualized with high sensitivity due to high uptake.

High liver and bowel uptake of ^18^F-PSMA-1007 has been reported. This was also found in the present group of patients. Since isolated liver metastases occur infrequently and bowel metastases very rarely, we consider that the high uptake in these organs would not cause a marked deterioration in the sensitivity of the tracer. In the present study, the sensitivity of ^18^F-PSMA-1007 PET/CT seemed to be independent of the GS. Prior studies using ^68^Ga-PSMA-11 have only shown a probable tendency for higher detection rates with increasing GS [3].

The lack of histopathology was a limitation of the present study. Therefore, we cannot exclude false-positive lesions, although the images were analysed by physicians with long experience of PET imaging, especially PCa imaging with PSMA-targeted radioligands.

### Conclusion

^18^F-PSMA-1007 PET/CT can detect recurrent PCa in a high percentage of patients with biochemical relapse. The probability of a pathological ^18^F-PSMA-1007 PET/CT seems to be high even in patients with a low PSA level of ≤0.5 ng/ml, which may have a significant impact in the further clinical management of patients. Prospective controlled trials are mandatory to validate these data.
